# Manipulating one-way space wave and its refraction by time-reversal and parity symmetry breaking

**DOI:** 10.1038/srep29380

**Published:** 2016-07-08

**Authors:** Yin Poo, Cheng He, Chao Xiao, Ming-Hui Lu, Rui-Xin Wu, Yan-Feng Chen

**Affiliations:** 1 School of Electronic Science and Engineering, Nanjing University, Nanjing 210093, China; 2 National Laboratory of Solid State Microstructures & Department of Materials Science and Engineering, Nanjing University, Nanjing 210093, China; 3 Collaborative Innovation Center of Advanced Microstructures, Nanjing University, Nanjing, 210093, China

## Abstract

One-way transmission and negative refraction are the exotic wave properties founded in photonic crystals which attract a great attention due to their promising applications in photonic devices. How to integrate such two phenomena in one material or device is interesting and valuable. In this work, we theoretically and experimentally demonstrate that one-way electromagnetic space wave can be realized by means of two-dimensional magnetic photonic crystals. Simultaneously breaking the time-reversal and parity symmetries of the magnetic photonic crystals designed, we observe oblique incident space wave propagating one-way in the magnetic photonic crystals with positive or negative refraction occurring at interfaces, which can be manipulated upon the incident angle and operating frequency. Our work may offer a potential platform to realize some exotic photoelectronic and microwave devices such as one-way imaging and one-way cloaking.

Energy and momentum are the two fundamental issues in physics. Correspondingly in electromagnetics, the frequency, wave vector and their relationship are important degrees to characterize energy flow and phase propagation of electromagnetic (EM) waves or light^1^,^2^. Meanwhile, time, space (as the Fourier transform pairs of frequency and wave vector) and their symmetries are often used to control the light propagation in designing artificial EM materials. Particularly, breaking continuous spatial translation symmetry into periodic one (or saying discrete spatial translation symmetry) would reshape the relation between the frequency and wave vector, forming band structures of photonic crystals (PCs)^3^,^4^,^5^. Similarly, the photonic time crystal can also be expected via breaking time translational symmetry^6^,^7^. By manipulating such translation symmetry, we can switch light on and off in both energy and momentum spaces as we like. On the other hand, time-reversal (T) symmetry, spatial inversion symmetry or parity (P) in momentum space and corresponding symmetry breakings play profound roles in controlling nonreciprocal energy flow and asymmetrical phase propagation. Associated this theme with band structure concept, lots of one-way propagation models were designed by utilizing PC with broken T or/and P symmetries^8^,^9^,^10^,^11^,^12^,^13^,^14^,^15^,^16^,^17^,^18^,^19^,^20^,^21^. For example, the chiral energy transportation from gapless edge modes in T symmetry-breaking PC^8^,^9^,^10^,^11^, the directional diffraction in P symmetry-breaking PC^12^,^13^, and the one-way transmission in both P and T symmetry-breaking PC^14^,^15^,^16^ were elaborately designed and demonstrated.

In addition to the frequency and wave vector aforementioned, the relationship between energy flow and phase propagation directions has also earned much attention especially since the negative refraction (energy and phase processing opposite propagating directions) proposed by Veselago^22^ was experimentally demonstrated. By controlling these physical quantities of waves, PCs and metamaterials have successfully made a revolution in material science and engineering to access much exotic functionality, such as superlens^23^,^24^,^25^,^26^,^27^ and invisible cloak^28^,^29^,^30^. Inspired by the recent studies on one-way optical propagation^8^,^9^,^10^,^11^,^12^,^13^,^14^,^15^,^16^,^17^,^18^,^19^,^20^,^21^, how to realize, especially experimental verifications of one-way negative refraction or even one-way cloaking is of great interests^31^,^32^.

In this work, we theoretically and experimentally demonstrate the realization of one-way space waves in a two-dimensional composite magnetic photonic crystal (MPC). Both one-way negative and positive refraction are observed under different excitations. Under the external bias magnetic field (BMF) and the special configuration of the unit cell, the composite MPC breaks P symmetry and T symmetry simultaneously. The symmetries breakings result in incident EM wave beams transmit through the MPC slabs in one incident direction but are totally reflected at the opposite direction, forming a one-way space wave with negative or positive refractions. The study shows such one-way negative or positive space wave can be manipulated by the incident angle and operating frequency. The corresponding experimental results verify such one-way space wave and are in a good agreement with the theoretical predictions. This work offers theoretical and implementary base for promising applications such as EM diode, one-way superlens and cloaking.

## Results and discussion

### Photonic crystal design

The one-way space wave appears in a system processing T and P symmetry breakings simultaneously. In this system, a pair of counter-propagating Bloch wave vectors, 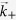 and 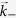, at an angular frequency *ω* yields


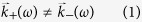


**Figure 1:**
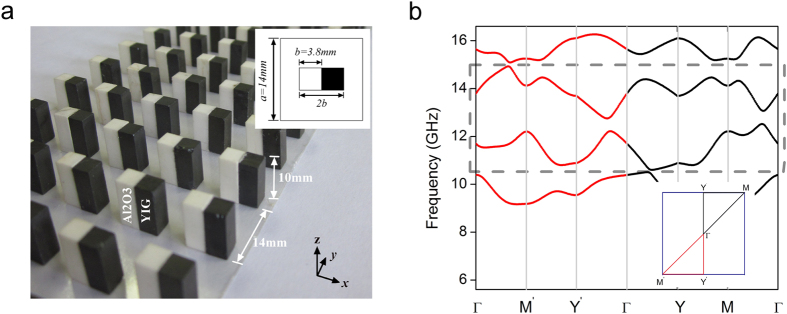
Symmetry-breaking MPC. (**a**) The MPC sample (5 × 16 cells) is arranged in square lattice. Each cell is composed of adjacent YIG pillar (black) and ceramics squared rods (white). The inset is a close look of the fabricated sample. The lattice constant is *a* = 14 mm. The YIG and ceramic squared rods have the same geometry size with side length of 3.8 mm and height of 10 mm. (**b**) Bandstructures of the MPC (from 8 GHz to 16 GHz). A 500 Oe BMF is applied along +z axis. Rectangled region represents the typical one-way propagation bands. The inset shows high-symmetry counter-directions of reciprocal space used in bandstructures.

One possible realization of one-way space wave is using PCs. It is easy to break T symmetry of PC by using magneto-optical materials^33^, and break P symmetry by introducing asymmetry spatial configuration in each unit cell. Following this line, we designed a composite PC for which a close look is displayed in Fig. 1a. The MPC has two square rods in each unit cell: the alumina (Al_2_O_3_, 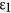 = 9.8-j0.005) and the ferrite (YIG, ε_2_ = 15.26-j0.003) pillars. The two types of pillars have the same geometry size and are under a BMF along +z-axis. Here, the method to break P symmetry by the composite of two different materials is flexible to manipulate the extent of symmetry breaking and design a desirable one-way refraction. For the configuration, the formal eigen-equation of the MPC derived from Maxwell equations can be written as,


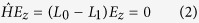


where *L*_0_ is the main operator and *L*_1_ is the perturbation one. In transverse magnetic (TM) mode in which electric field is polarized along rods axis (+z axis), the operator *L*_0_ and *L*_1_ for the ferrite rods are of the form as


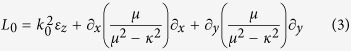



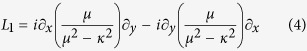


where *μ* and *κ* are the elements of YIG’s tensor permeability and satisfy, respectively, 
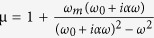
, 
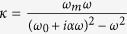
. Here, *ω*_0_ = γH is the resonance frequency, γ is gyromagnetic ratio, H is BMF, *ω*_m_ = 4πM_s_ is the characteristic frequency, ε_z_ is z-axis permittivity component and *k*_0_ is the wave vector in vacuum. Equation (3) indicates T symmetry breaking of the MPC since gyrotropic perturbation *L*_1_ is pure imaginary. Meanwhile, the asymmetric structure of the unit cell along x-axis results in 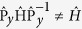 (*P*_*y*_ is the y-component of parity operator) which induces P symmetry broken along k_y_ direction. Thus, both T and P symmetry breakings are realized along y-axis. The symmetry breakings eventually cause solutions of Eq. (2) not coming out in pair, which is the base of one-way space wave. It should be noticed that the MPC still keeps P symmetry along *k*_x_ direction, therefore only for a pair of counter-incident EM waves with opposite signs *k*_y_ components can excite one-way space wave.

Besides the broken T and P symmetries, asymmetric bulk bandstructure is another necessary condition to realize one-way transportation. It requires that one incident wave meet passband of MPC and its counter-incident wave vector meet bandgap at the same time. Figure 1b gives the band structures in two counter-directions (ΓY′M′Γand ΓYMΓ). The band curves display asymmetric shape between ΓY and ΓY′ directions indicating 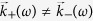. In the typical asymmetric frequency range (10.6 GHz~15 GHz) marked by the dashed box in Fig. 1b, some bumps and pits present on the opposite sides of Brillouin zone center corresponding to Bloch wave vectors pointing to opposite directions. Therefore, in the frequency range, oblique incident EM wave will pass through the MPC in one direction and be totally reflected in the opposite direction, which can be directly verified by the transmission measurements.

### One-way refraction

**Figure 2:**
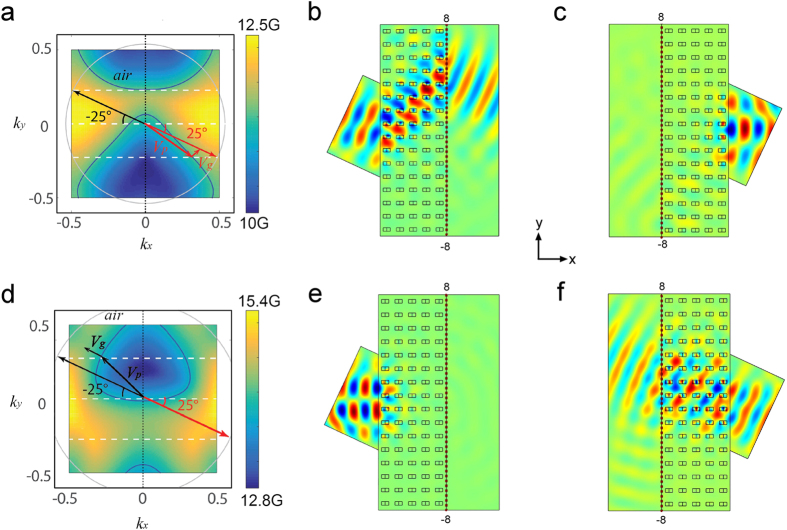
One-way negative/positive refraction in symmetry-breaking MPC. (**a**,**d**) EFS maps of the second and third band (Fig. 1b) in the first Brillouin zone. In the two figures, the white curves and gray circles are the EFSs of the MPC and air at operating frequencies f_1_ = 11.89 GHz and f_2_ = 13.86 GHz, respectively. The red and black arrows represent the wave vector of the incident wave with incident angels +25° and −25°. The indicating arrows labeled as group velocity (V_g_) and phase velocity (V_p_) show the directions of the transmitted wave in the MPC. The simulated electric field distributions at the incident angles of (**b**,**e)** +25° from left-up; (**c**,**f**) −25° from right-down, respectively.

Equivalent frequency surface (EFS) of the MPC is calculated to illustrate one-way refraction of the space wave in detail. Figure 2a displays the EFS of the second band in Fig. 1b, which covers the frequencies from 10.6 GHz to 13.4 GHz. The contours are asymmetric along *k*_y_ axis, consistent with band structure shown in Fig. 1b. The contours are strongly bended with a whole shift-up which is totally different from that of PCs with P and T symmetry. Such contours result in the one-way refraction at the interface between the MPC and air background. Suppose the interface of MPC is along y-axis, and incident wave obliquely projects on the MPC interface from two opposite directions as shown in Fig. 2a. The red and black arrows represent the wave incidence at +25° and −25°, respectively. The white and gray circles are the contours of the MPC and air at frequency f_1_ = 11.9 GHz. We see that the wave incidence at angle +25° can meet the boundary condition at the interface where the contours of air and MPC have the same y-components of wave vector, however the boundary condition is not satisfied at opposite incident angle. This indicates at f_1_ EM wave can transmit through the MPC with positive incident angle but will be totally reflected at the opposite incident angle, i.e. the one-way propagation. The direction of the transmitted or refracted wave is decided by the gradient of the contours of the MPC, i.e. the direction of group velocity V_g_ of the refracted wave. If the directions of the refractive and incident waves are in the same side of the normal to the interface, the refraction is a negative refraction; otherwise it is a positive refraction^16^. From Fig. 2a, we can identify the refraction at the MPC interface is a negative refraction. Figure 2b,c further give numerical results of electric field distributions in the case of ±25° incidence. They show a clear one-way negative refraction and the angle of negative refractive is about −42°. In addition to the negative refraction, the one-way space wave in the MPC can be positive refraction too. Figure 2d plots the EFS of the third band in Fig. 1b. The EFS shows a big difference from that of the second band. Take frequency f_2_ = 13.86 GHz as an example, its contour is totally located in the upper part of the contour map. EM wave incident from air with positive incident angle +25° (red arrow) no longer intersects with the contour of MPC, instead the wave with negative angle −25° (black arrow) does. In this circumstance, the MPC only allows the latter incident wave to pass through. From the EFS, it can be seen that the direction of refracted wave and incident wave lay on the two sides of the normal, representing a positive refraction. Figure 2e,f are the simulated electric field distributions at operating frequency f_2_. The figures show the EM wave is totally reflected when the incident angle equals to +25°. As a comparison, in the case of −25° incidence, EM wave can easily go through the MPC accompanied with a positive refraction happening at the interface, showing a one-way positive refraction.

### Observation of one-way refraction

**Figure 3:**
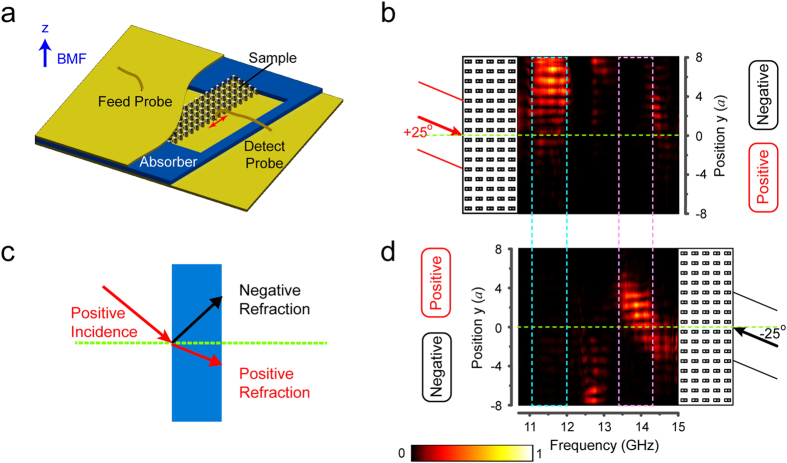
Experimental setup and measurement. (**a**) Schematic picture of experimental setup. The MPC sample is sandwiched between two aluminum plates (yellow) and surrounded by the absorber (blue). In the measurement, the oblique incident angle is achieved by tilting the MPC sample. (**c**) Schematic of refraction for positive incident angle. The green dashed line is the normal line distinguishing positive and negative refraction. For the case that the detect probe slides along the MPC’s interface, positive and negative refraction correspond to the outgoing wave center laying below and above the normal. (**b**,**d**) are experimental emergent wave field distribution spectra with a pair of counter incident angles. (**b**) +25° from left-up and (**d**) −25° from right-down. Bright and dark color represent the large and small power flow, x axis is operating frequency, and y axis is the position of out-going interface (from −8*a* to 8*a*). The dotted lines are the beam centers of incident wave (normal). There are two typical one-way regions: one-way negative refraction from 11.1 GHz to 11.9 GHz with +25° incidence; one-way positive refraction from 13.4 GHz to 14.1 GHz with −25° incidence.

Experimentally, an MPC sample in an array of 5 × 16 cells was fabricated as shown in Fig. 1a. The experimental setup is schematically displayed in Fig. 3a, where the sample MPC slab is sandwiched between two metallic plates and surrounded by absorber. A plane wave polarized along +z-axis obliquely impinges on the sample. The emergent wave out of the other side of the MPC slab is detected by a movable detector sliding along the MPC’s interface. The detector moves from bottom to top along the dashed lines shown in Fig. 2b,c,e,f, which covers 16 unit cells from −8*a* to 8*a (a* is the lattice constant).

The type of refraction occurring at the interface of the MPC can be distinguished by the position of the wave beam center of the emergent wave at the out-going interface of the MPC. As schematically illustrated in Fig. 3c, for positive incidence, the center of the emergent wave beam will lay above the green dashed line if the refraction is negative, otherwise the wave beam center will appear below the dash line for the positive refraction. In experiments, the wave beam center is determined by recording the electric field distribution at the out-going interface of the MPC. Figure 3b,d plot the experimental results of the electric field distribution at the MPC’s out-going interface as a function of frequency and detector’s positions when EM wave incidents at ±25°.

For +25° incidence shown in Fig. 3b, the strong electric field distributes above the beam center of the incident wave (the green dotted line) in the frequency range of 11.1 GHz to 11.9 GHz boxed by the cyan dashed lines. The field intensity is very small below the green dot line. The results indicate the refraction at the interface of MPC is negative by comparing the schematic of negative refraction in Fig. 3c. In contrast, in Fig. 3d where the incident angle is −25°, the field distribution is almost zero in the same frequency range. The huge difference of emergent wave beam distribution between two opposite wave incidences proofs the space wave in the MPC sample is one-way. Differently, in the frequency range from 13.3 GHz to 14.1 GHz which is marked by the purple box in Fig. 3b,d, the measured electric field distribution obviously shows the one-way space wave in the MPC has a positive refraction at its interfaces. The experiments are consistent with the results obtained from EFS analysis shown in Fig. 2d.

**Figure 4:**
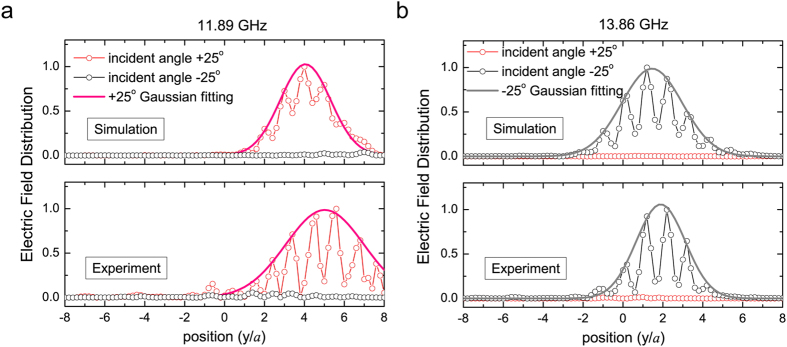
Out-going field distribution along the interface of MPC (from −8a to 8a along y axis) at two operating frequencies. (**a**) 11.89 GHz and (**b**) 13.86 GHz. In each figure, the experimental data (lower panel) agree well with simulation results (upper panel).

Based on the measured field distributions at the out-going interface of the MPC, we can further quantitatively retrieve the refractive angle of one-way refraction. Figure 4 plots the normalized field distribution along the MPC’s outgoing interface. In the figure, the field oscillation is due to diffraction of the rods. Using Gaussian fitting (the bold line), we obtain an envelope of the emergent wave beam. The maximum value of envelop is approximately considered to be the center of emergent wave at the interface of MPC. As displayed in the lower panel of Fig. 4a, the maximum of field magnitude appears at y = +5*a* corresponding to a refraction angle of −45° at 11.89 GHz. As a comparison, the upper panel gives the simulated field distribution at the interface and the fitting curve of the field envelop. Same with the experiments the field distribution is also oscillating. The simulated center of emergent wave locates at y = +4.2*a* (corresponding to refractive angles −40°), which is in a good agreement with that retrieved from experimental data. For one-way positive refraction, Fig. 4b plots the detailed field distributions and Gaussian fitting envelop at 13.86 GHz. Only under −25° incidence, EM wave can transmit through the MPC with outgoing beam center at +1.5*a* in simulation (upper panel) and 1.8*a* in experiment (lower panel), corresponding to the refractive angle +19.8^o^ in simulation and +21.8^o^ in experiment, respectively. It should be noticed that the fluctuation difference between the simulations and experiments in Fig. 4 is mainly caused by the wave front of incident waves. Compared to the ideal plane wave in our simulations, the incident wave in experiment is excited by a line source locating in a wave channel with absorber side walls as shown in Fig. 3a. Such wave channel allows wave propagation in a broadband frequency range but will induce the wave front deviating from plane wave’s. In addition, since the outgoing wave front is sensitive to the detect position. The deviation between the simulation and experiment maybe also cause some difference.

### Relationship of one-way refraction with incident angle

**Figure 5:**
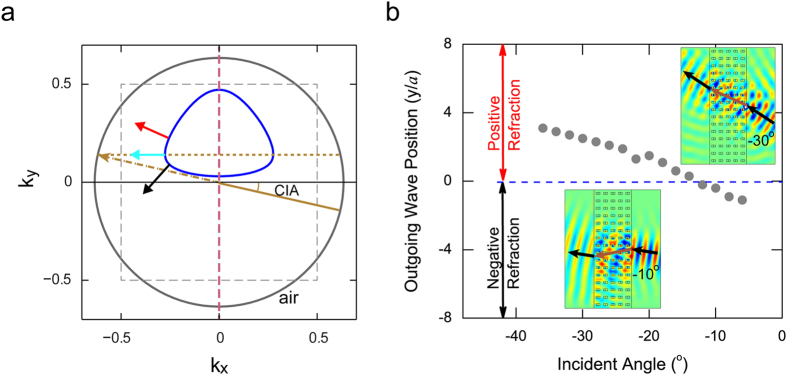
Incident angle-dependent refractive types. (**a**) The navy curve and grey circle are the EFSs of the MPC and air at frequency of 13.6 GHz. For negative incident angle, there exists a CIA (the brown arrow) which corresponds to a zero refracted wave (cyan arrow). Red and black arrows show the directions of the positive and negative refracted wave. (**b**) The simulation result of incident angle-dependent negative/positive refraction at 13.6 GHz. The CIA is −12.5°. When the incident angle is larger than CIA, a positive refraction will appear. On the contrary, a negative refraction will appear. The insets are two typical refractive field distributions.

The observed one-way refraction in such symmetry-breaking MPC is not limited to a specified incident angle, but exists in a large range of incident angles. Referred in Fig. 2a,d, the one-way refractions have a close relationship with the incident angles. As an example, Fig. 5a draws the contours of the MPC and air at the frequency 13.6 GHz. The contour of MPC strongly bends as a fillet triangle-shape locating in the upper plane of Brillouin zone. Therefore, only the EM waves coming from the right-down may excite the space wave in MPC. Due to the special shape of the EFS, there exists a critical incident angle (CIA) for which the refracted wave beam within the MPC has zero refractive angle, that is the direction of refracted wave beam is normal to the interface. Deviate from this CIA, the refraction will be positive one (red arrow) or negative one (black arrow). Figure 5b draws the simulated center position of the outgoing wave beam versus incident angle from −40° to −5°. The CIA is about −12.5° at 13.6 GHz. The figure shows once the incident angle is larger than CIA, the refraction will be positive, otherwise it will be negative. The insets plot two typical field distributions for positive and negative refraction when incidence angle is −30° and −10°, respectively. The one-way space wave occurring in a big range of incident angle might provide a convenient way to the practical applications such as one-way cloaking and one-way focusing.

## Conclusion

In summary, we have theoretically and experimentally demonstrated that one-way space wave can be realized using a specially designed MPC. Here, T symmetry is broken by using magneto-optical material YIG, and P symmetry breaking is achieved by the composite of two different materials (YIG and alumina) which is more flexible to manipulate the extent of symmetry breaking for desirable one-way refraction compared to pure geometry modulation^16^. Furthermore, our work is focused on experimental realization of one-way refraction. All the numerical calculations and simulations fully take the effects of dispersion and loss effect into account, which match the experimental results very well. In certain frequency range and incident angles, the EM waves transmit through the MPC in one direction and are prohibited in the opposite direction. Such one-way space waves can be excited in large incident angles and wide frequency range, and the refraction occurring at the interface of the MPC could be negative, zero or positive, which can be manipulated by the incident angle and operating frequency. The realization of one-way space wave offers promising applications in optoelectronic devices such as optical isolator, providing a practical way toward one-way focusing and one-way cloak.

## Methods

### Materials and sample fabrication

Theoretically, all the models with broken P and T symmetries may support one-way phenomenon. Any dielectric can be used in design. Due to our experimental setup limitations which requires working frequency under 15 GHz, bias magnetic field less than 1000 Oe and sufficient number of elements within the uniform bias magnetic field provided by the Helmholtz coils with diameter of 260 mm, commercial YIG ferrite and common alumina ceramics (low loss and large permittivity compared to vacuum) were used here. The saturation magnetization of YIG ferrite 4πM_s_ = 1884 Gauss was measured by vibrating sample magnetometer (VSM). The relative permittivity of YIG and alumina at microwave frequencies was measured by transmission/reflection method and they are 15.26-j0.003 and 9.8-j0.005 respectively. The YIG and alumina were machined into squared rods. In fabrication, the YIG rods and the ceramic rods were stuck on one of the metal plate of the parallel plated waveguide to construct an array in a size of 5 × 16 unit cells.

### Experimental setup and measurement

As schematically shown in Fig. 3a, the measurement setup includes vector network analyzer (VNA) Agilent E8363A, a long parallel plate waveguide, and Helmholtz coils with inner diameter 26 cm. The sample was sandwiched in the parallel waveguide and far away from the excitation probe which is connected to port 1 of VNA. The sample was placed with its normal line off the center line of the waveguide to make the excited plane wave obliquely incident on the interface of the sample. The emergent wave beam was obtained by measuring the electric field distribution at the outgoing interface of the sample by a movable detect probe connected to port 2 of VNA. The center of the emergent wave beam was determined by maximum magnitude of the field distribution along the interface.

### Band structure and EFS calculations

The band structure and EFS map were calculated by using commercial software COMSOL MULTIPHYSICS with RF module. The solution steps were first using eigenvalue solver to obtain initial values of the Eq. (3) and then with nonlinear solve to get eigen frequencies with wave number changing from k = 0 to 0.5.

## Additional Information

**How to cite this article**: Poo, Y. *et al*. Manipulating one-way space wave and its refraction by time-reversal and parity symmetry breaking. *Sci. Rep.* **6**, 29380; doi: 10.1038/srep29380 (2016).
